# The promise and limits of patient-reported outcomes in molecular tumor board-guided care: toward a framework for patient-centered precision oncology

**DOI:** 10.1038/s41698-026-01719-0

**Published:** 2026-10-29

**Authors:** L. Gräßel, S. Yacob, C. Strantz, E. Prochaska, T. Debertshäuser, AA. Rogge, S. Bäcker, F. Prasser, P. Unberath, J. Wehrle, S. Jäger, M. Boerries, AL Illert, U. Lührig, M. Wolfien

**Affiliations:** 1 Department of Hematology and Medical Oncology, University Medical Center Göttingen, Göttingen, Germany; 2 Department of Medicine I, Medical Center-University of Freiburg, Faculty of Medicine, University of Freiburg, Freiburg, Germany; 3 Medical Informatics, Friedrich-Alexander-Universität Erlangen-Nürnberg, Erlangen, Germany; 4 Institute for Medical Informatics and Biometry, Medical Faculty and University Hospital Carl Gustav Carus, TUD Dresden University of Technology, Dresden, Germany; 5 Charité - Universitätsmedizin Berlin, Berlin Institute of Health @ Charité, Berlin, Germany; 6 Center for Patient-Centered Outcomes Research, Charité – Universitätsmedizin Berlin, Corporate Member of Freie Universität Berlin and Humboldt-Universität Zu Berlin, Charité Platz 1, Berlin, Germany; 7 Department of Psychosomatic Medicine, Charité – Universitätsmedizin Berlin, Corporate Member of Freie Universität Berlin and Humboldt-Universität Zu Berlin, Berlin, Germany; 8 Medical Center for Information and Communication Technology, Universitätsklinikum Erlangen, Friedrich-Alexander-Universität Erlangen-Nürnberg, Erlangen, Germany; 9 SRH Fürth University of Applied Sciences, Fürth, Germany; 10 Data Integration Center, Medical Center-University of Freiburg, Faculty of Medicine, University of Freiburg, Freiburg im Breisgau, Freiburg, Germany; 11 Institute of Medical Bioinformatics and Systems Medicine, Medical Center-University of Freiburg, Faculty of Medicine, University of Freiburg, Freiburg, Germany; 12 Department I of Internal Medicine, Center for Integrated Oncology Cologne, University Hospital Cologne, Cologne, Germany; 13 Center for Scalable Data Analytics and Artificial Intelligence (ScaDS.AI Dresden/Leipzig), Dresden, Saxony, Germany

**Keywords:** Cancer, Health care

## Abstract

Patient-reported outcomes (PROs) capture symptoms, functioning, quality of life, treatment burden and priorities directly from patients. Their integration into Molecular Tumor Board (MTB)-guided care remains limited and particularly challenging, as recommendations often involve off-label therapies, sparse evidence and complex benefit-burden trade-offs. In this perspective, we propose a four-domain framework encompassing measurement, data integration, decision support and governance to guide longitudinal monitoring and patient-centered learning.

## Integrating patient-reported outcomes into molecular tumor board-guided care: rationale and scope

Molecular tumor boards (MTBs) have emerged as a key component of personalized medicine and precision oncology, generating highly individualized treatment recommendations^1^. They are particularly relevant for patients with advanced cancers, rare malignancies, exhausted guideline-based options, or potentially actionable molecular alterations^2,3^. These recommendations often involve complex benefit-burden trade-offs and require the integration of molecular, clinical, and therapeutic information.

Despite the central role of MTBs in treatment decision-making, the patient perspective is not yet systematically incorporated into MTB workflows. To date, PROs are primarily collected in clinical trials and research settings, while their integration into routine cancer care and precision oncology remains limited^4^. Here, we argue that integrating patient-reported outcomes (PROs) into MTB-guided care is both clinically necessary and methodologically feasible. PROs can serve two complementary purposes: supporting patient-centered monitoring of off-label therapies during follow-up, and contributing to the systematic generation of patient-centered evidence that may inform future MTB deliberations.

This manuscript is intended as a Perspective article rather than a systematic review. It draws on the authors’ interdisciplinary expertise in oncology, MTBs, PROs, medical informatics, interoperability, data integration, and precision oncology infrastructure. The initiatives, standards, and implementation challenges discussed are intended to illustrate key requirements for PRO integration into MTB workflows. Based on these considerations, we propose a conceptual framework that can guide future research, technical implementation efforts, and stakeholder co-design.

## Advancing patient-centered care in precision oncology: the case for integrating pros into molecular tumor boards

### PROs capture what clinical documentation misses

PROs have become a key element of patient-centered care by systematically incorporating the patient perspective into clinical assessment. PROs are defined as “any report of the status of a patient’s health condition that comes directly from the patient without interpretation of the patient’s response by a clinician or anyone else”^5,6^. By integrating these into routine care, patients can participate more actively in their treatment journey, while clinicians gain access to information that is often only partially reflected in routine documentation or clinical trial endpoints.

PROs complement rather than replace clinician-assessed outcomes. In oncology, clinician-reported toxicity grading, such as the common terminology criteria for adverse events (CTCAE), remains essential for standardized safety assessment^7,8^. However, it may not fully capture the frequency, severity, timing, or day-to-day impact of symptoms as experienced by patients^9^. PROs add a structured patient perspective on treatment impact, symptom burden, and health-related quality of life^10^, which is especially relevant when treatment decisions require balancing potential benefit against tolerability and patient-defined goals.

### Standardized PROMs turn patient experience into comparable data

PROs are commonly collected through Patient-Reported Outcome Measures (PROMs), standardized instruments that capture and quantify patient experiences. Developed through considerable international efforts, PROMs enable reproducible assessment across studies and care settings^11^. Table 1 summarizes established PROMs, their domains, and their potential relevance to MTB-guided care. PROMs differ in scope and target population, and their clinical value depends on selecting appropriate instruments, defining meaningful assessment time points, and integrating results into clinical workflows to achieve interpretable and actionable data.

**Table 1:** Selection of patient-reported outcome measures (PROMs) and their applicability in the molecular tumor board context

Instrument	Type	Structure	Domains captured	Relevance in MTB context
PRO-CTCAE^8,62,63^	Symptom-specific	Fixed item library, single items; no total scale	Symptomatic adverse events (frequency, severity, interference)	Pragmatic toxicity monitoring of off-label therapies; not indication-specific
EORTC QLQ-C30^64,65^	Cancer-specific	Fixed multi-item questionnaire (core module)	Global health status, functional scales, symptom scales	Health-related quality of life trajectory assessment; broad applicability across MTB patient population
FACT-G^66,67^	Cancer-specific	Fixed multi-item questionnaire (4 domains)	Physical, social, emotional, functional well-being	Health-related quality of life trajectory assessment; broad applicability across MTB patient population
PROMIS^68,69^	Generic (item banks)	IRT-calibrated item banks; configurable short forms and CATs	Modular: fatigue, pain, physical function, anxiety, depression, etc.	Flexible, modular structure suited to heterogeneous MTB population
EQ-5D-5L^70^	Generic (health utility)	Fixed questionnaire with 5 dimensions + VAS	Mobility, self-care, usual activities, pain/discomfort, anxiety/depression	Suitable for cross-indication comparison and health utility estimation; limited sensitivity for symptom-level monitoring
ICHOM Standard Sets^71,72^	Disease-specific	Composite set (mix of external instruments)	Condition-dependent; combines PROMs with clinical outcomes	Relevant where disease-specific sets exist; often no direct Set available for rare or highly off-label MTB indications

Instrument selection for MTB-guided therapy monitoring requires consideration of validation scope, domain coverage, and the structural absence of indication-specific PROMs for off-label therapies. *PRO‑CTCAE* patient‑reported outcomes version of the common terminology criteria for adverse events, *EORTC QLQ‑C30* European Organization for Research and Treatment of Cancer core quality of life questionnaire, *FACT‑G* functional assessment of cancer therapy—general, *PROMIS* patient‑reported outcomes measurement information system, *EQ‑5D* EuroQol 5‑dimension questionnaire, *ICHOM* international consortium for health outcomes measurement, *IRT* item response theory, *CAT* computerized adaptive testing, *VAS* visual analogue scale

Evidence from clinical trials, systematic reviews, and real-world implementations suggests that incorporating PROs into routine care can strengthen symptom monitoring, patient-clinician communication, supportive care, and treatment management^12–14^. While evidence for an overall survival benefit remains inconsistent, PROs have demonstrated benefits across a range of other patient-centered outcomes, including toxicity burden, physical functioning, and health-related quality of life. However, effective PRO implementation requires defined clinical response pathways, technical integration, patient engagement, and organizational capacity.

### MTBs concentrate on situations where patient experience matters

MTB recommendations frequently involve off-label therapies for which clinical trial data, including context-specific PROMs, are limited^15,16^. As a result, treatment decisions are often made under considerable uncertainty regarding both clinical benefit and treatment burden. In this setting, close treatment monitoring becomes particularly important from both a clinical and ethical perspective, as ongoing assessment is needed to determine whether treatment continues to provide meaningful value for the individual patient.

In addition, many patients discussed in MTBs have advanced, heavily pretreated malignancies and face treatment decisions in late-line settings^17,18^. In these situations, therapeutic decisions increasingly require balancing potential benefit against symptom burden, functional status, quality of life, and individual patient goals. Understanding how patients experience treatment, therefore, becomes particularly important and raises the question of how to incorporate this perspective into MTB-guided care.

### Why PROs are needed in MTB-guided care

The rationale for integrating PROs into MTBs is neither to introduce another standalone data source nor to replace established forms of molecular and clinical evidence. Instead, PROs address the distinct clinical need to systematically capture how patients experience MTB-guided care and its consequences. While molecular and clinical data inform treatment recommendations, PROs provide information on how treatment affects symptoms, functioning, quality of life, and patient priorities.

PROs can serve two complementary purposes in the MTB context. First, they can support longitudinal monitoring following an MTB recommendation by systematically capturing patient-reported symptoms, functional limitations, treatment burden, and preferences. Making this information visible during follow-up visits enables evaluation of the tolerability and feasibility of the current therapeutic strategy.

Second, PROs can contribute to evidence generation. MTB cases are often highly individualized and may approach n-of-1 treatment situations^19^. Patient cohorts are typically small and heterogeneous, limiting the generalizability of conventional evidence across molecular alterations, tumor entities, treatment lines, and drug combinations^1^. This creates a particular challenge for evaluating the clinical benefit of MTB-recommended therapies. Von Hoff *et al*. proposed using a ratio of progression-free survival under MTB-recommended therapy compared to the preceding treatment as a proxy for individual treatment benefit^20^. However, such metrics remain limited in capturing the full spectrum of patient-relevant outcomes. Systematically collected PROs may complement these approaches by adding information on the real-world consequences of MTB-guided therapies from the patient perspective. However, linking PROs with molecular, clinical, and treatment data does not resolve the inherent sparsity of n-of-1-like treatment situations. If all dimensions were used simultaneously for stratification, they might even further fragment already small patient groups. PROs should therefore not be positioned in a fixed hierarchical order with tumor type or molecular alterations, but rather considered as an orthogonal patient-centered dimension whose relevance depends on the intended question^21^. While assessments of treatment efficacy continue to require molecular and clinical context, selected PRO domains, such as symptom burden, functioning, or treatment tolerability, may support broader comparisons across otherwise heterogeneous treatment contexts and contribute to hypothesis-generating learning.

Although MTBs are not necessarily the simplest setting for PRO implementation, they represent a particularly high-value use-case. Disease-specific oncology pathways, organ boards, or routine systemic treatment settings may offer larger patient populations, more standardized PROM schedules, and clearer clinical escalation pathways^6^. Nevertheless, MTBs concentrate highly individualized treatment decisions under conditions of uncertainty, which makes a structured understanding of patients’ experiences especially important. Realizing the potential of PROs to provide this additional dimension of information, however, requires standardized measurement, technical integration, and defined response pathways; otherwise, PRO collection risks becoming an additional documentation burden with limited clinical value.

### Maria’s case: illustrating PRO-enabled MTB care

To illustrate a possible future PRO-enabled MTB workflow, we constructed a fictional case based on the collective clinical experience of the participating physicians. The protagonists do not correspond to individual persons.

Imagine a future MTB workflow in which Maria, a 47-year-old patient with advanced breast cancer, receives an off-label therapy based on her tumor’s molecular profile. Several weeks after treatment initiation, she reports increasing fatigue, nausea, and joint pain through an electronic PRO (ePRO) system. Her treating physician reviews these reports during follow-up visits, where they complement routine clinical assessment. Maria’s symptoms may affect her therapy adherence, daily functioning, and quality of life, yet may not be fully reflected in her physician’s routine documentation.

In a mature PRO-enabled MTB workflow, Maria’s patient-reported information would be displayed alongside molecular findings, treatment history, laboratory values, imaging results, clinician-assessed toxicity, and current treatment goals^22^. The ePRO system would employ predefined thresholds to flag clinically relevant symptom trajectories. In Maria’s case, her functional decline could indicate an increasing treatment burden that warrants closer clinical evaluation. This would prompt her treating physicians to reevaluate whether the current therapy remains tolerable and aligned with Maria’s priorities, or whether additional supportive measures or treatment adjustments are needed.

At the same time, Maria’s experience would not only inform her own care. It could also contribute to a growing evidence base on how patients experience MTB-guided therapies beyond established standard-of-care treatment pathways. Over time, aggregated data across clinically meaningful treatment contexts may help characterize patterns of tolerability and patient-reported benefit associated with specific therapeutic strategies.

Maria’s fictional case illustrates both functions of PROs in MTB-guided care: supporting patient-centered treatment monitoring and contributing to the generation of patient-centered evidence. Realizing this potential, however, requires more than simply collecting patient-reported data. The following sections, therefore, discuss the key requirements and challenges for integrating PROs into MTB workflows.

## From potential to practice: requirements and challenges for PRO integration in MTBs

### PROs require defined action pathways to create clinical value

The clinical value of PROs in MTBs depends not on data collection alone, but on whether patient-reported information informs clinical action. Evidence from recent Danish ePRO trials suggests that collecting PROs without sufficient organizational capacity to review and act on it may not translate into measurable clinical benefits^23^. By embedding PROs into treatment monitoring and response pathways, patient-reported symptoms, treatment burden, functional limitations, and preferences become visible and actionable. This can support more informed shared decision-making while empowering patients to contribute actively to decisions about their care^24,25^. Additionally, PROs can provide longitudinal feedback on treatment burden and quality of life, enriching clinical discussions and enabling earlier supportive interventions where appropriate^26,27^.

However, collecting PRO data is insufficient if no mechanisms exist to translate PRO signals into appropriate clinical responses. A high fatigue score, for example, does not automatically imply dose reduction, treatment discontinuation, or referral to supportive care; rather, it requires clinical interpretation and contextualization. Effective implementation, therefore, needs predefined thresholds, escalation rules, documentation standards, and clear assignment of clinical responsibility^11^. Without these elements, PRO collection risks increasing the documentation burden without improving care. Seamless integration of PROs into clinical workflows is thus a prerequisite for meaningful and actionable PRO use^28^.

Standardized and interoperable PROs can further facilitate consistent data collection across institutions, which enhances research through quality assurance and cross-site learning. From a health-system perspective, PROs may also contribute to more efficient care by enabling earlier symptom management, reducing unnecessary interventions and streamlining care coordination. However, these potential benefits depend on implementation costs, staffing resources, workflow integration, and sustained clinical use^29^.

### Missing data, cross-sectoral coordination and digital inequities are major implementation challenges

Despite their potential value, implementing PROs in MTBs and oncological patient care in general faces significant hurdles. A particularly important limitation is the missing PRO data^30^. Even in prospective clinical studies with highly motivated patients and study teams, PRO completion rates may decline over time, and patterns of missingness may be related to disease progression, treatment toxicity, hospitalization, cognitive or physical impairment, digital fatigue, or death. Missing PRO data were not merely a technical limitation but may themselves be clinically informative^31^. MTB workflows should therefore define how missing data are monitored, interpreted, and documented, and whether missingness itself should trigger clinical follow-up.

This challenge is amplified in routine MTB care by cross-sectoral collaboration. Patients are referred to an MTB by their treating physician, who may be located within the same hospital or in an external practice. At some centers, externally referred patients constitute a substantial share of MTB cases (~30%), and an increase in the number^32,33^. For these patients, treatment is often supervised by the external physician, while the MTB recommendation is generated at a specialized center. Acquiring PRO-based data, integrating them into MTB documentation systems, and ensuring appropriate follow-up requires cross-sectoral workflows and digital tools that connect patients, external treating physicians, and MTB centers.

Accessibility must also be considered when setting up PRO collection workflows. ePRO systems may unintentionally disadvantage patients with limited digital literacy, language barriers, cognitive impairment, severe disease burden, limited access to devices, or low trust in digital health systems^34–37^. Therefore, PRO systems should be designed with accessibility, multilingual support, usability, and alternative reporting pathways in mind, ideally with the involvement of patient organizations and patient representatives to ensure that implementation reflects patient needs rather than solely institutional requirements.

### Ethical, regulatory, technical and governance requirements must be addressed early

Integrating PROs into MTB workflows is not only a clinical challenge but also a challenge in terms of governance and infrastructure. Regulatory and ethical requirements, including informed consent, data privacy, access control, and secondary use of patient-reported information, must be addressed from the outset. These considerations become particularly important when PROs are linked to other sensitive data like molecular profiles, treatment histories, imaging data, and outcomes, or when they are used in AI-supported analytics. Within frameworks such as the General Data Protection Regulation (GDPR)^38^ and the European Health Data Space (EHDS)^39^, patient autonomy, transparency, data minimization, explainability, and trust become central implementation conditions.

Technical barriers present additional challenges. Interoperability, data security, semantic standardization, and scalability remain major barriers to adoption^22,40^. PROMs may be implemented using local identifiers, different versions, heterogeneous assessment schedules, or proprietary data formats. Even when interoperability standards such as HL7 FHIR for data exchange^41^ or OMOP for standardized observational data were adopted^42^, local mapping, questionnaire versioning, and semantic harmonization require substantial effort. Without shared definitions and reusable data models, PRO data risk remains siloed, limiting comparability across institutions and hindering integration into broader precision oncology infrastructures.

Successful PRO implementation in MTBs, therefore, ultimately depends on coordinated and effective stakeholder engagement. Clinicians require training, time, and streamlined tools to interpret PRO data without increasing cognitive burden^22^. Patients need accessible tools, transparent information on data use, and confidence that their reports will be acknowledged and acted upon^43^. Technology providers, policymakers, payers, researchers and industry partners must support interoperable, secure, and sustainable infrastructures while avoiding proprietary lock-in, to preserve data portability, clinical flexibility, and patient trust^44^. Addressing these governance, technical, and organizational requirements early will be essential for translating PRO collection into meaningful and sustainable clinical value for MTB patients.

Given the conceptual and implementation-focused nature of these challenges, the following sections outline a framework for integrating PROs into MTB workflows and discuss the associated methodological, technical, and organizational considerations.

## A framework for PRO integration into MTB workflows

**Fig. 1:**
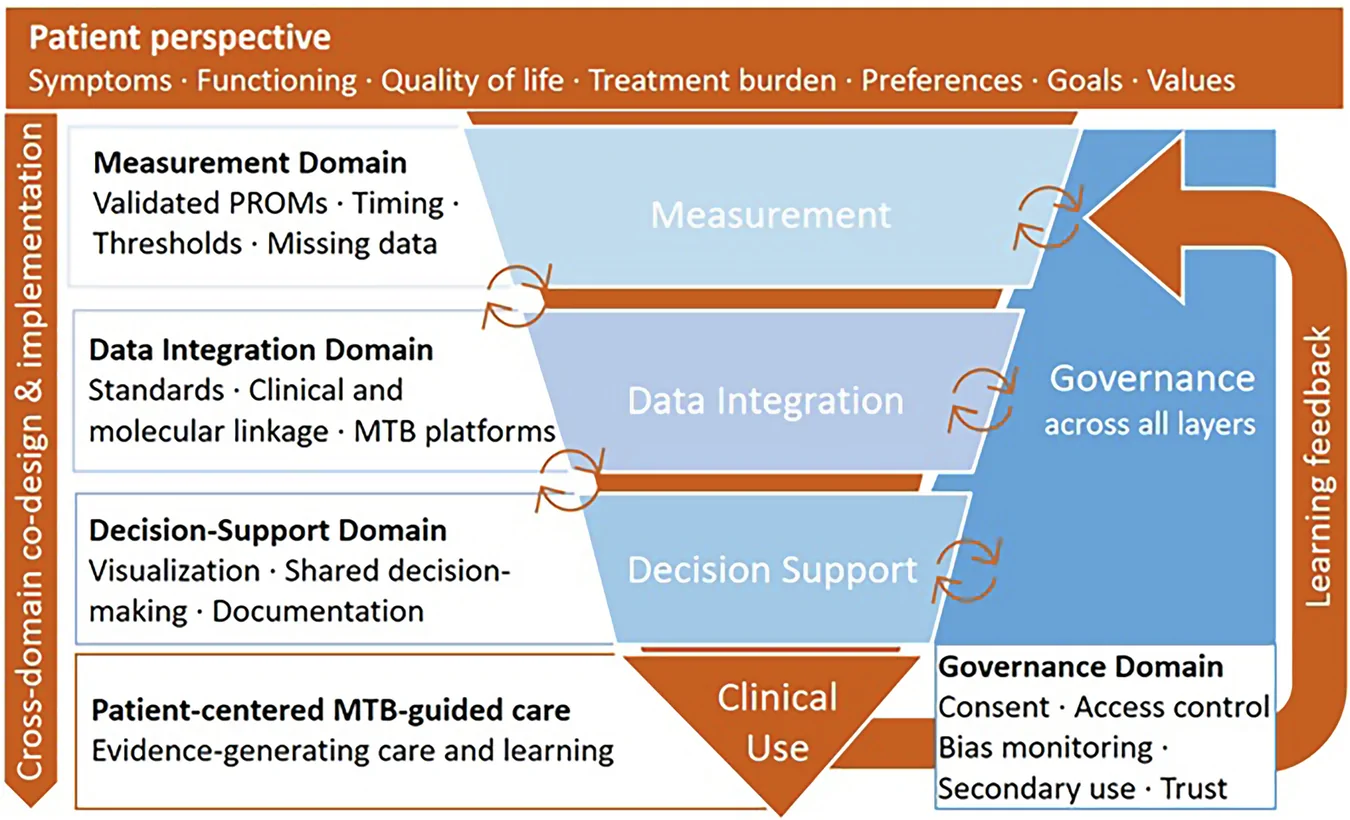
Interdependent four-domain framework for PRO-enabled Molecular Tumor Board-guided care. The framework comprises measurement, data integration, decision support, and governance as interconnected rather than sequential domains. The patient perspective spans the framework and encompasses symptoms, functioning, quality of life, treatment burden, preferences, goals, and values, emphasizing that PROs are intended to incorporate patients’ lived experience and priorities into clinical care rather than function solely as an additional data source. Governance acts across all domains and shapes measurement, access, interoperability, clinical use, and secondary use from the outset. Cross-domain co-design and implementation support adaptation to clinical and organizational contexts, while bidirectional interactions between the domains reflect their mutual dependencies. Clinical use supports patient-centered MTB-guided care and evidence-generating care and learning, with learning feedback informing subsequent measurement, integration, decision-support, and governance processes.

PRO integration into MTB-guided care serves two connected but distinct purposes: therapy-accompanying monitoring of MTB-guided recommendations and evidence-generating learning across patients and institutions. Both require more than simply collecting questionnaire responses. Patient-reported information must be captured with valid instruments, integrated with molecular and clinical data, translated into interpretable decision-support formats, and governed through clear rules for responsibility, consent, access and secondary use. We therefore propose a dynamic four-domain framework for PRO integration into MTB workflows comprising measurement, data integration, decision-support, and governance. Figure 1, therefore, represents these domains as interconnected rather than sequential components of the framework.

### Measurement: PROs must be measured using valid, feasible, and interpretable instruments

The first domain concerns which patient-reported information is collected, when it is measured and how it can be interpreted. PRO integration into MTBs should rely on validated instruments rather than locally developed questionnaires. Depending on the clinical context, these may include generic health-related quality of life instruments, cancer-specific PROMs, symptom-focused instruments, or treatment-specific modules. Established frameworks such as EORTC questionnaires, PROMIS item banks, PRO-CTCAE, EQ-5D, and ICHOM outcome sets (see also Table 1) provide important foundations for standardized measurement. However, no single instrument will be sufficient for all MTB contexts due to heterogeneity in tumor entities, treatment context, molecular profile and patient goals. Without valid, context-appropriate and interpretable measurement strategies, PRO data risk becomes difficult to compare and apply across MTB scenarios.

### Data integration: PROs must be connected to the clinical and molecular context

The second domain concerns how PRO data are connected to the clinical, molecular, and treatment information required for MTB-guided care. The value of PROs depends on their efficient technical and semantic integration to achieve accessibility within clinical information systems, tumor registries, molecular reporting systems or MTB platforms. In MTB-guided care, patient-reported burden should be interpreted alongside molecular findings, treatment history, laboratory values, imaging results, clinician-assessed toxicity, and treatment goals. Without this linkage, PROs remain isolated questionnaire outputs rather than clinically usable information.

Open interoperability standards such as HL7 FHIR (for structured exchange of questionnaire definitions and responses) and OMOP (for harmonized observational data models) can support data exchange and secondary use^41,45^. However, interoperability not only depends on technical standards, but also on transparent and consistent mapping of PROM definitions, response options, scoring rules, and assessment time points. Achieving such interoperability at scale further requires commitment from software vendors and technology providers. Sustainable PRO integration cannot be achieved by academic centers and healthcare institutions alone. Vendors developing patient-facing applications, hospital information systems, tumor documentation platforms, molecular reporting solutions, and MTB-support software should support vendor-neutral interfaces, open APIs, and modular architectures that enable PRO data to move across organizational and technical boundaries. Without such openness, PRO data risk becomes trapped within proprietary systems, limiting interoperability across clinical and precision oncology infrastructures.

In practice, this requires PRO data to flow seamlessly between patient-facing tools, hospital information systems, tumor documentation platforms, MTB-support software, such as MTB-cBioPortal^46,47^ and KnowledgeConnector^48^, or further clinical process models^49,50^. This is particularly important for multicenter precision oncology networks, where comparable PRO data from MTB-guided therapies could support quality assurance, real-world evidence generation and cross-institutional learning. Beyond this, such standardized data infrastructures may also provide a foundation for future AI-supported or patient-similarity approaches that analyze accumulated molecular, clinical and patient-reported data^35^,^36^. However, these applications should remain exploratory unless validated for clinical decision support^51^.

### Decision-support: PROs must be translated into actionable clinical information

The third domain concerns how PRO information is translated into clinically meaningful and actionable decision support. In the MTB context, decision support operates at two connected but distinct levels: patient-specific treatment monitoring and adjustment during an MTB-guided therapy, and integration of selected PRO summaries into MTB documentation, reassessment, and future learning. This distinction is important because most patient-reported symptoms require follow-up in the treating pathway rather than immediate deliberation by the full MTB.

For patient-specific monitoring, PROs should be presented as concise longitudinal summaries highlighting relevant trends in symptoms, functioning and treatment burden to the treating physician and their healthcare team. Worsening fatigue, pain, nausea, or physical function requires clinical interpretation, and alerts should trigger predefined follow-up pathways. Depending on context, this may include initiation of supportive care measures, medication review, psycho-oncological support, treatment adaptation, communication with the external treating physician, or reevaluation of treatment goals. Such pathways not only facilitate timely and consistent responses to clinically relevant PRO signals but may also reduce the cognitive burden of clinicians by providing structured guidance to manage complex and often unfamiliar MTB-guided treatment situations^52,53^. Clear allocation of responsibility is essential, particularly in cross-sectoral cooperation on externally referred MTB cases. Without such actionable workflows and clearly assigned responsibilities, PRO dashboards risk adding information without improving patient care or clinical decision making^28^. Empirical evaluations of PRO integration in MTB-specific workflows remain scarce. Future studies should prospectively assess clinical utility, patient acceptability, alert burden, time required for review, cross-sectoral communication needs, and effects on treatment monitoring and patient-centered outcomes.

At the MTB workflow level, PRO summaries can add patient-centered context to molecular and clinical findings, treatment history, clinician-assessed toxicity, and current treatment goals. Such summaries can document whether patient-reported burden affected treatment feasibility and adherence, or prompted reassessment of an MTB-guided recommendation. Decision support, therefore, refers to contextualization rather than automation: PROs can enrich deliberation and follow-up, but they do not replace clinical judgment or molecular evidence.

### Governance: PROs must be used responsibly and transparently

The fourth domain concerns the ethical, legal, and organizational conditions required for responsible and trustworthy use of PRO data. As PRO information becomes linked to molecular and clinical datasets, it forms part of highly sensitive, data-intensive precision oncology infrastructures. This raises requirements for clear standards, transparent governance, and harmonized regulatory frameworks^54^, particularly regarding consent, data access, secondary use, algorithmic bias, explainability, and patient autonomy.

In this context, data ownership should be understood less as a narrow property claim and more as a question of patient control, stewardship, access rights, and transparency. Many patients may be willing to share PRO data for research or to help future patients in similar clinical situations, particularly when facing rare molecular constellations or off-label treatment decisions. However, such solidarity-based data use must remain voluntary, understandable, and revocable where possible^55^. Consent models should therefore clearly distinguish between the use of PROs for direct care, quality assurance, and secondary research. They should also explain who can access the data, for which purposes, whether data may be linked with molecular and clinical information, and what safeguards are in place to prevent misuse or unintended re-identification^56^.

If PRO data are used in AI-supported analytics or patient-similarity approaches, governance must also address algorithmic bias and representativeness^57^. Missing PRO data, digital exclusion, language barriers, disease severity, and small MTB subgroups can systematically shape which patients' experiences enter the data infrastructure. Models trained on such data may therefore underrepresent patients with high symptom burden, limited digital access, or rare clinical contexts. AI-supported analyses should consequently remain exploratory unless prospectively validated, monitored for bias, and embedded in transparent clinical accountability structures.

Beyond regulatory compliance, governance must ensure that PRO use remains aligned with patient trust. Patients should understand why their data are collected, how PRO information informs clinical care, whether secondary use for research is possible, and what feedback they can expect. Sustainable implementation further requires collaboration with patient organizations to define acceptable workflows, feedback mechanisms, access rights, and governance structures. Without transparent governance and trust-centered implementation, PRO integration risks becoming an institutional data collection exercise rather than a genuinely patient-centered component of precision oncology.

Taken together, these interdependent domains continuously shape one another, as governance requirements influence measurement, access, interoperability, and clinical use from the outset, while decision-support needs can determine which PRO domains should be collected and how they need to be integrated and presented. Evidence generated through clinical use should, in turn, feed back into subsequent decisions on measurement, data integration, decision-support design, and governance, creating an iterative learning cycle rather than a unidirectional implementation pathway.

## Implementation prerequisites: integrating PROs into precision oncology infrastructures

Recent European evidence in precision cancer medicine demonstrates that the implementation of advanced molecular diagnostics depends on coordinated organizational, technical, legal and data infrastructures as well as interdisciplinary collaboration^58^. We argue that PRO integration requires a similar approach. Rather than creating a parallel data stream, patient-reported information should be embedded within existing precision oncology data ecosystems. Several national and international initiatives already address complementary aspects of this challenge, ranging from clinical networks and outcome standardization to interoperable data infrastructures and governance frameworks (Table 2).

**Table 2:** Selected national and international infrastructures and initiatives relevant to PRO integration in precision oncology

Initiative	Core mission	Relevance for PRO–MTB integration	Transferable lesson
DNPM / ZPM German Network for Personalized Medicine/Centers for Personalized Medicine	Establish networked structures for personalized oncology and MTB-based care across participating centers	Provides clinical and organizational structures in which molecular, clinical, and potentially patient-reported information could be jointly considered	PRO integration should be embedded into existing precision oncology pathways rather than developed as a parallel system
PM4Onco Precision Medicine for Oncology	Develop and harmonize digital and methodological approaches for precision oncology across German MTBs	Offers a concrete environment for testing user-centered visualizations, MTB documentation, and integration of PROMs into oncology workflows	Shared implementation blueprints can support harmonization while allowing adaptation to local infrastructure and maturity
MII/DIZ Medical Informatics Initiative/Data Integration Centers	Build interoperable infrastructures for integrating clinical and research data across university hospitals	Support standardized capture, harmonization, and secondary use of PROs together with clinical and molecular data	PROs require institutional data infrastructures, not only patient-facing apps
PCOR-MII Patient-Centered Outcomes Research in the Medical Informatics Initiative	Establish cross-disease structures for collecting and using patient-centered outcomes	Provides methodological and infrastructural links between routine care, research, and patient-reported data	PRO implementation should be scalable beyond oncology and should use reusable standards
H2O Health Outcomes Observatory	Promote standardized patient-centered outcome collection and value-based healthcare in Europe	Demonstrates how PROs can be embedded into broader outcome observatories and international benchmarking efforts	International harmonization is needed to avoid isolated local PROM implementations
EHDS European Health Data Space	Create a European framework for health data access, exchange, and secondary use	Provides a policy and governance context for cross-border health data use, including patient-generated and patient-reported data	PRO integration must address governance, trust, interoperability, and secondary-use rules from the beginning
ICHOM International Consortium for Health Outcomes Measurement	Define standardized outcome sets across diseases, including patient-reported domains	Supports selection and harmonization of PROMs relevant to disease-specific and cross-disease care pathways	Standardized outcome sets can improve comparability, but local clinical workflows still determine actionability

This table summarizes selected German and European initiatives that are relevant to the integration of patient-reported outcomes (PROs) into precision oncology and molecular tumor board (MTB) workflows

These initiatives demonstrate that the prerequisites for PRO-enabled MTB workflows are already emerging across the precision oncology ecosystem^59–61^. Clinical networks, outcome frameworks, interoperable data infrastructures, and governance initiatives each address different components of the challenge. The remaining challenge is therefore not to create entirely new structures, but to connect and adapt these existing elements for PRO-enabled MTB workflows. Ongoing implementation activities can provide practical guidance on how this transition may be achieved.

### From framework to implementation: a pragmatic roadmap

The proposed framework is informed by ongoing implementation activities within precision oncology infrastructures represented by the authors. Within the three-year PM4Onco project, participating sites are developing local roadmaps for integrating PROs into existing Data Integration Centers and MTB-related workflows. Implementation maturity currently varies across sites: some already have relevant data and technical components available or are approaching operational integration, whereas others are still establishing the necessary organizational and technical prerequisites. Rather than mandating a uniform architecture, PM4Onco provides shared technical implementation blueprints that can be adopted and adapted to local infrastructure and readiness (https://github.com/PM4Onco).

A pragmatic implementation pathway includes local readiness assessment, selection and co-design of suitable PROMs and assessment schedules, technical integration into existing clinical and MTB-support infrastructures, pilot implementation of longitudinal monitoring and predefined response pathways, and prospective evaluation of usability, clinical utility, patient acceptability, and workflow burden. Components shown to be feasible can subsequently be harmonized across participating sites, providing a pathway from local implementation to scalable PRO integration across precision oncology networks.

### Stakeholder responsibilities for implementation

Implementing these steps depends on coordinated action across multiple stakeholder groups. Measurement, integration, decision support, and governance cannot be established by any single actor alone but require collaboration across clinical, technical, regulatory, research, and patient-facing domains (Table 3).

**Table 3:** Stakeholder responsibilities for PRO-enabled molecular tumor board-guided care

Stakeholder	Primary responsibility	Concrete implementation task
MTB teams and clinicians	Clinical actionability	Define when PROs are reviewed, who responds, and how PRO findings enter MTB recommendations
Patients and patient organizations	Relevance, acceptability, accessibility	Co-design PROM schedules, reporting interfaces, consent language, and feedback loops
Medical informatics and data infrastructure teams	Interoperability and data quality	Implement FHIR/OMOP-compatible data capture, semantic mapping, and linkage to molecular and clinical data
Industry and software vendors	Scalable technical solutions	Provide vendor-neutral interfaces, usable dashboards, secure APIs, and integration into existing clinical systems
Regulators and policymakers	Governance and incentives	Clarify reimbursement, secondary-use rules, data protection requirements, and alignment with EHDS/GDPR
Researchers and registries	Evidence generation	Evaluate clinical utility, missing data, equity effects, and patient-centered outcomes across sites

This table outlines the main stakeholder groups required for integrating PROs into molecular tumor board (MTB) workflows. It links each group to a primary responsibility and a concrete implementation task

## Conclusion

Molecular tumor boards represent a particularly high-value yet challenging, use-case for integrating PROs into precision oncology. Their recommendations frequently involve off-label therapies, limited evidence, and highly individualized benefit-burden trade-offs, making an understanding of the patient perspective especially important. Although PROs cannot resolve the evidence limitations inherent to many MTB-guided treatment decisions, they can make treatment burden and consequences visible, enhance shared decision-making and enable patient-centered therapy management. When collected systematically and linked with molecular, clinical, treatment, and outcome data, PROs may additionally contribute to evidence generation in treatment settings where conventional clinical evidence remains sparse.

To support these complementary goals, we propose a four-domain framework encompassing validated measurement, interoperable data integration, clinically meaningful decision support, and trustworthy governance. Together, these domains provide a concept for integrating patient-reported information into MTB-guided care while ensuring clinical usability, technical interoperability, and patient trust.

Future work should evaluate which PROM domains are most relevant for MTB-guided therapies, how PROs can be integrated into existing precision oncology infrastructures, and how stakeholders can jointly develop sustainable implementation models. If these challenges are addressed successfully, PROs may help transform MTB-guided precision oncology from a predominantly molecularly informed approach into one that is simultaneously molecularly informed, genuinely patient-centered, and capable of learning from real-world patient experience.

## Data Availability

No original datasets were generated or analyzed for this Perspective article. All information discussed in this work is contained in the manuscript. No custom code or software was generated or used for this work.
